# Supplementary material for: Discovery of hexagonal ternary phase Ti2InB2 and its evolution to layered boride TiB
Source: Nat Commun. 2019 May 23;10:2284. doi: 10.1038/s41467-019-10297-8 (PMC6533257; doi:10.1038/s41467-019-10297-8)
Supplement: Supplementary file 1 — Supplementary Information [file 41467_2019_10297_MOESM1_ESM.pdf]

Supplementary Information

Discovery of hexagonal ternary phase Ti_2InB_2 and its evolution to layered boride TiB

Wang et al.

Supplementary Method

Synthesis of Ti_2InB_2: Since indium has a much lower boiling point (2072 ^{o}C) than that of Ti (2836 ^{o}C) and B (3927 ^{o}C), the compound cannot be grown from the melt. The synthesis using arc melting (under 600mbar extra pure Ar, purity >99.99995%) was failed, because In evaporate seriously before reaction. Therefore Ti_2InB_2 was prepared by using a solid-state reaction route in the present study. Still, the yield was very small, because Ti_2InB_2 competed with other impurity phases (mainly TiB_2 and Ti-In phases). In order to improve the yield, many experiment conditions: temperature effect, crucible effect, atmosphere effect, annealing effect and the initial composition ratio effect, were considered. (Supplementary Fig. 12-17 and Table 3)

The growth of Ti_2InB_2 is very sensitive to temperature, only intermediate temperatures (1100 ^{o}C to 1200 ^{o}C) work well for synthesis (Supplementary Fig. 12). Above 1200 ^{o}C, the target phase becomes thermodynamically less stable than other phases, while below 1000 ^{o}C, the growth was rather slow. Sample container also has a big influence on the synthesis (Supplementary Fig. 13 and Table 3). In a sealed stainless steel (SUS) tube, indium did not tend to react with other elements, and a lot of TiB_2 was produced. A sealed quartz tube with Mo foil covering the sample worked much better. The reaction atmosphere was also taken into account (Supplementary Fig. 14 and Table 3). When the tube was sealed with Ar gas inside, indium tended to react with the other elements and the formation of TiB_2 was suppressed; however, if the tube was sealed under vacuum, indium evaporated and covered the inner wall of the tube, leading to an indium loss and a high yield of the impurity phase TiB_2. The influence of the reaction atmosphere is mainly ascribed to the low triple point of indium (157 ^{o}C and 1 kPa), therefore, in order to suppress the vapor loss of indium, a sealed environment was chosen rather than an open environment with Ar gas flow. Annealing was not effective to improve the growth of Ti_2InB_2, but on the contrary, the yield of Ti_2InB_2 was gradually decreasing under annealing at different conditions (Supplementary Table 3). The influence of initial composition ratio of Ti, In and B was considered at the end. By increasing the amount of only Ti by 15%, the yield of Ti_2InB_2 was much improved and the yield of TiB_2 was largely decreased. However a further increase of Ti to 30% dramatically suppressed the growth of Ti_2InB_2. A similar behavior was observed when the initial amount of In was adjusted, and the results were summarized in the Supplementary Table 3. Surprisingly, when excess amount of both In and Ti were added at the initial step, the yield of TiB_2 monotonously decreased as the excess amount increased, and yield of Ti_2InB_2 reached a maximum value (Supplementary Fig. 15(a)). Finally, the obtained samples were washed by a diluted hydrochloric acid, and the impurity phases except TiB_2 were removed.

The open circuit voltages (OCVs) for an intercalation reaction involving Li^+ and Na^+ ions can then be estimated from the energy difference of charge/discharge process as following:

$$\text{OCV} \approx E(\text{TiB}) + E(\text{Li, Na}) - E(\text{TiBLi, TiBNa}) \qquad (1)$$

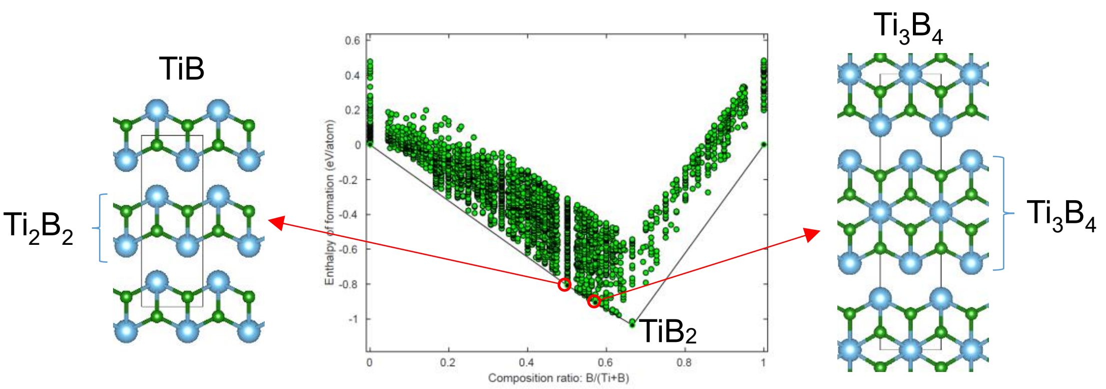

Supplementary Figure 1. Evolutionary structure search result of Ti-B system. Besides the known TiB_2 and TiB (*Pnma*), two layered structure TiB (*Cmcm*) and Ti_3B_4 were obtained.

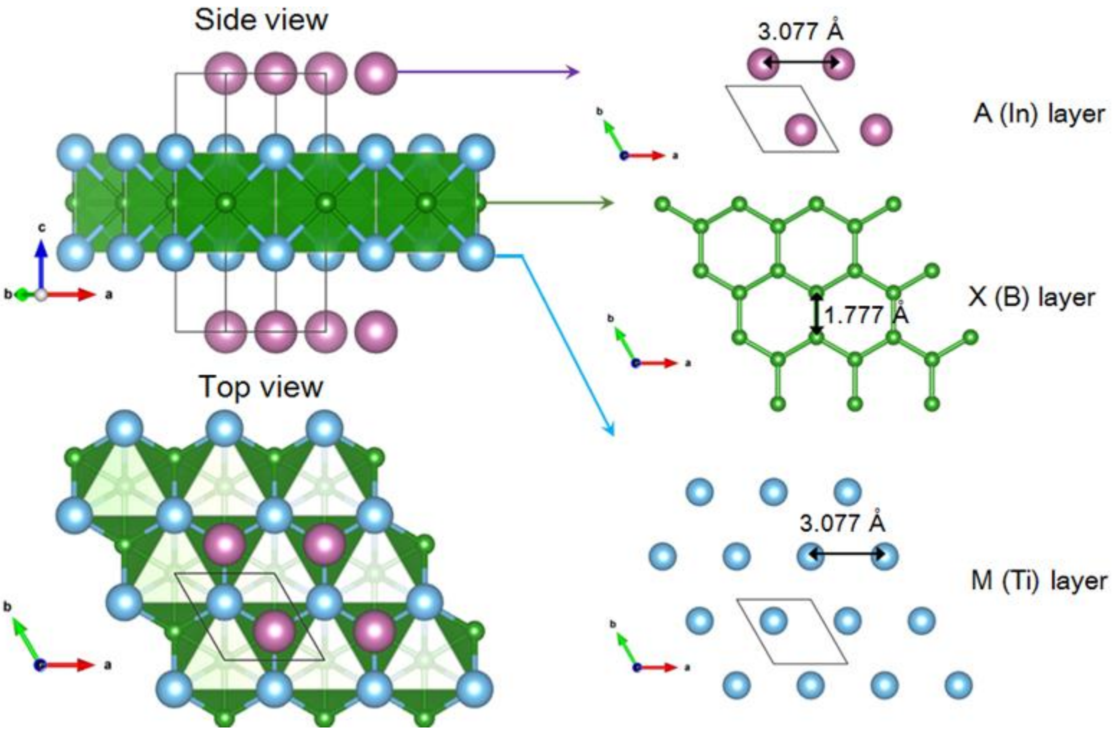

Supplementary Figure 2. Crystal structure of the predicted boron-containing MAX phase Ti_2InB_2 and the atom stacking manners of the M, A and X layers.

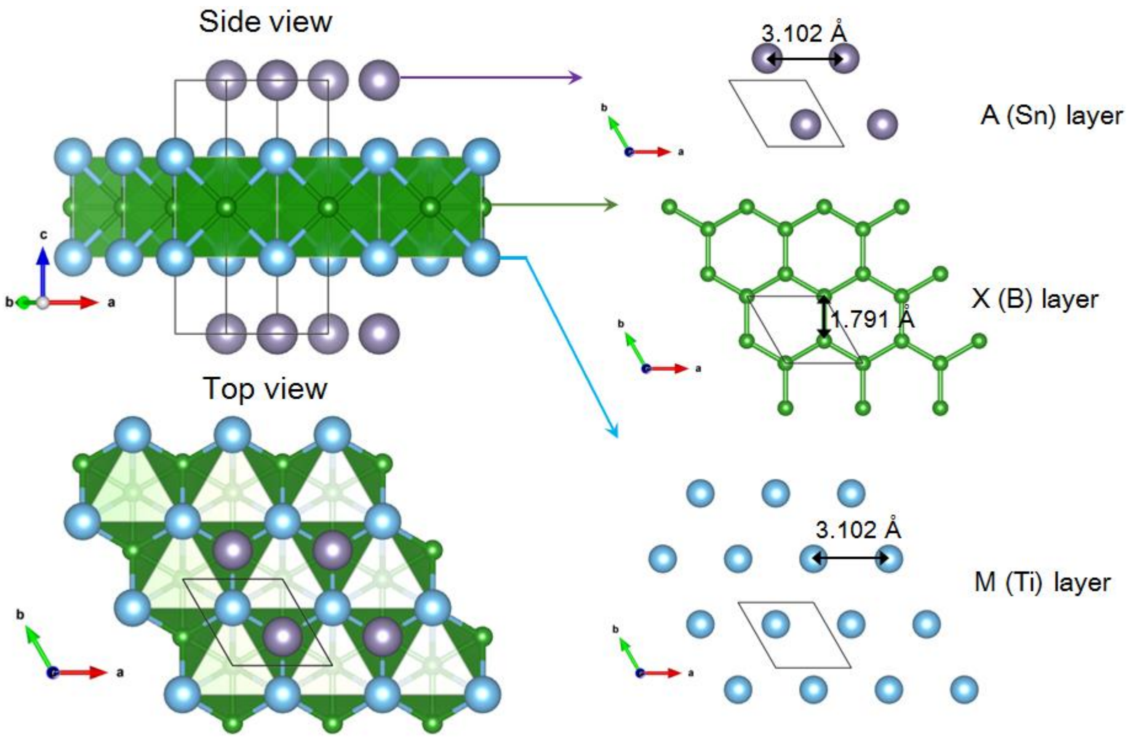

Supplementary Figure 3. Crystal structure of the predicted boron-containing MAX phase Ti_2SnB_2 and the atom stacking manners of the M, A and X layers.

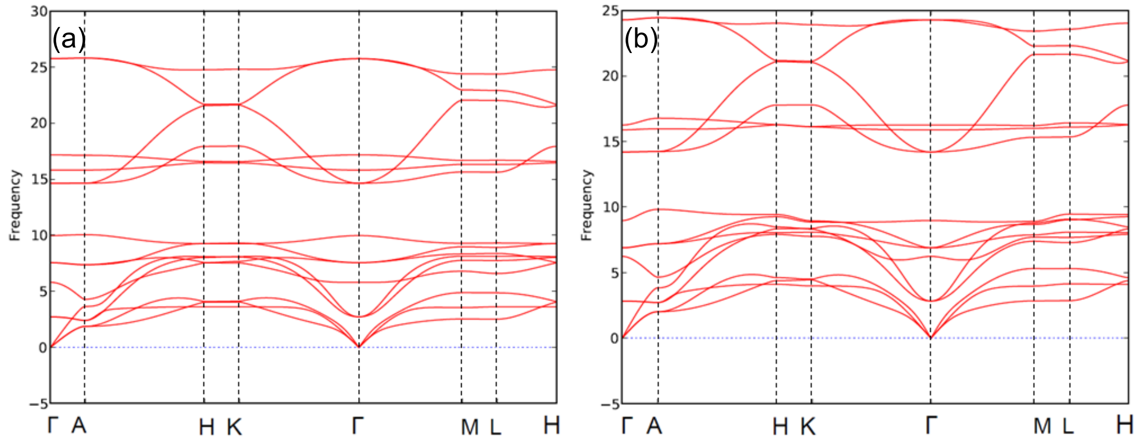

Supplementary Figure 4. Phonon band structures of the predicted (a) Ti_2InB_2 and (b) Ti_2SnB_2 structures at 1 atm pressure.

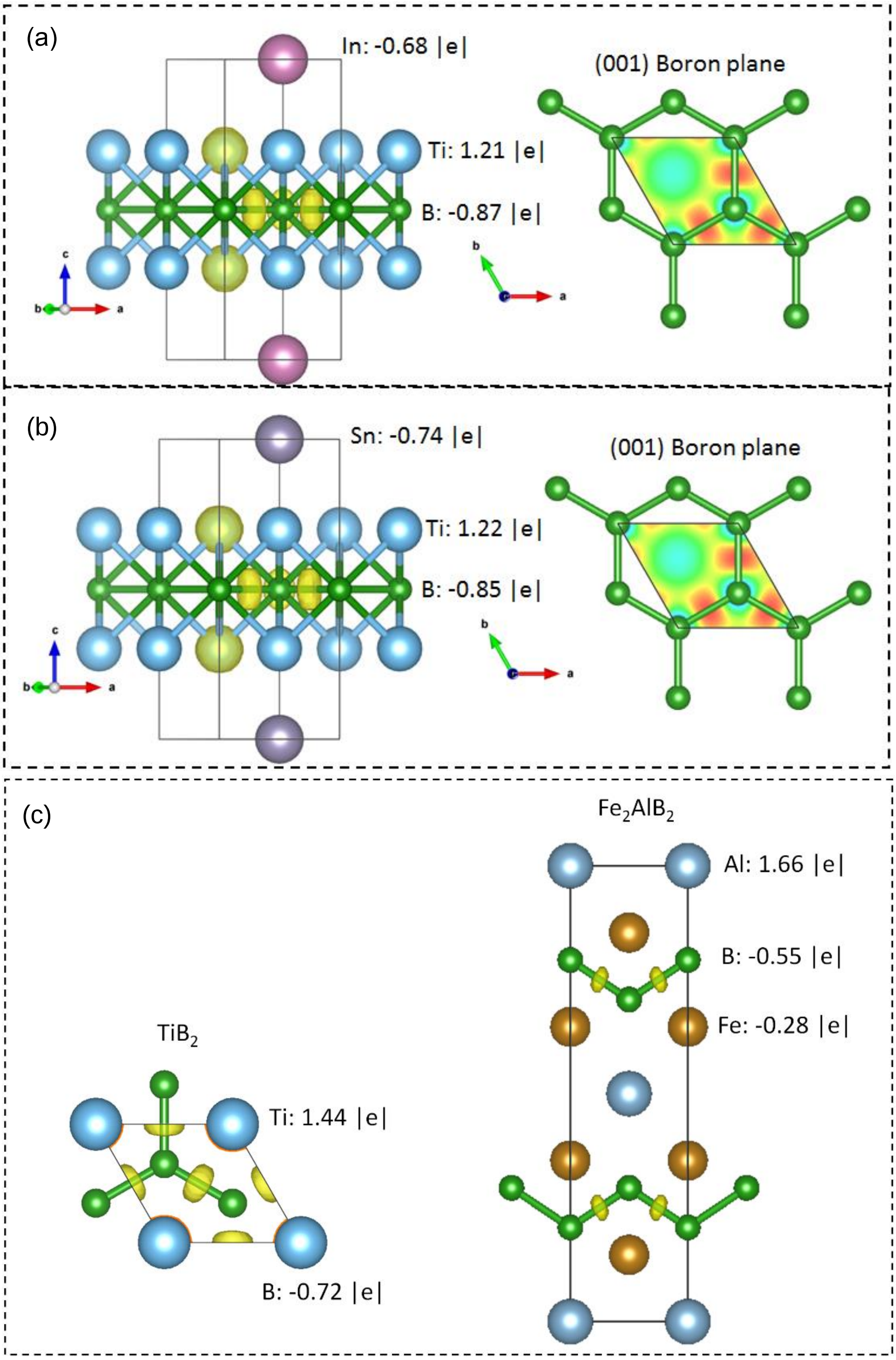

Supplementary Figure 5. Electron localization function (ELF) and Bader charge calculation results for the predicted (a) Ti_2InB_2 and (b) Ti_2SnB_2 structures in comparison with the situation in (c) TiB_2 and MAB phase Fe_2AlB_2.

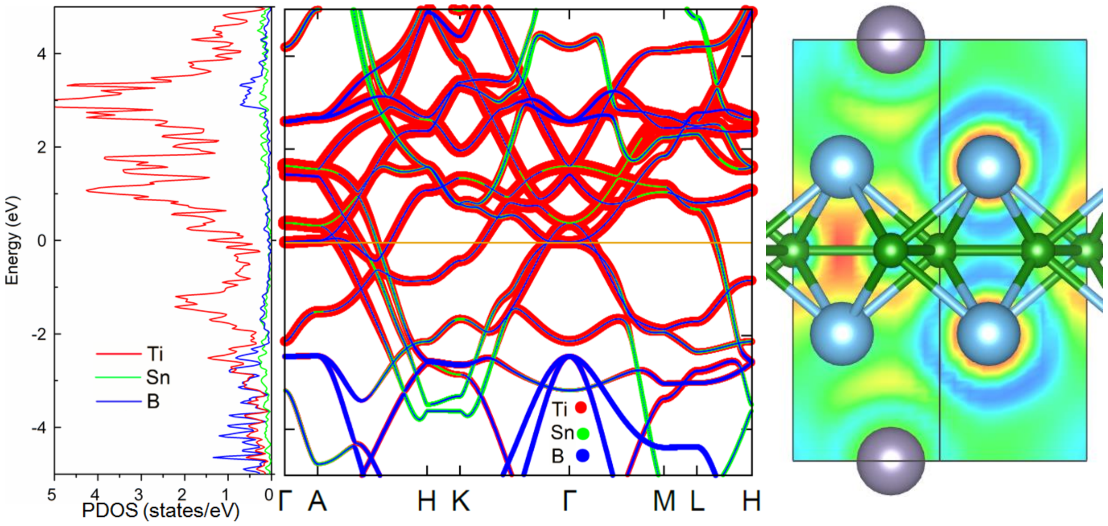

Supplementary Figure 6. The calculated electronic structures of Ti_2SnB_2. The projected density of states (DOS), projected band structures and electron localization function (ELF) for each structure are shown in the left, central and right regions.

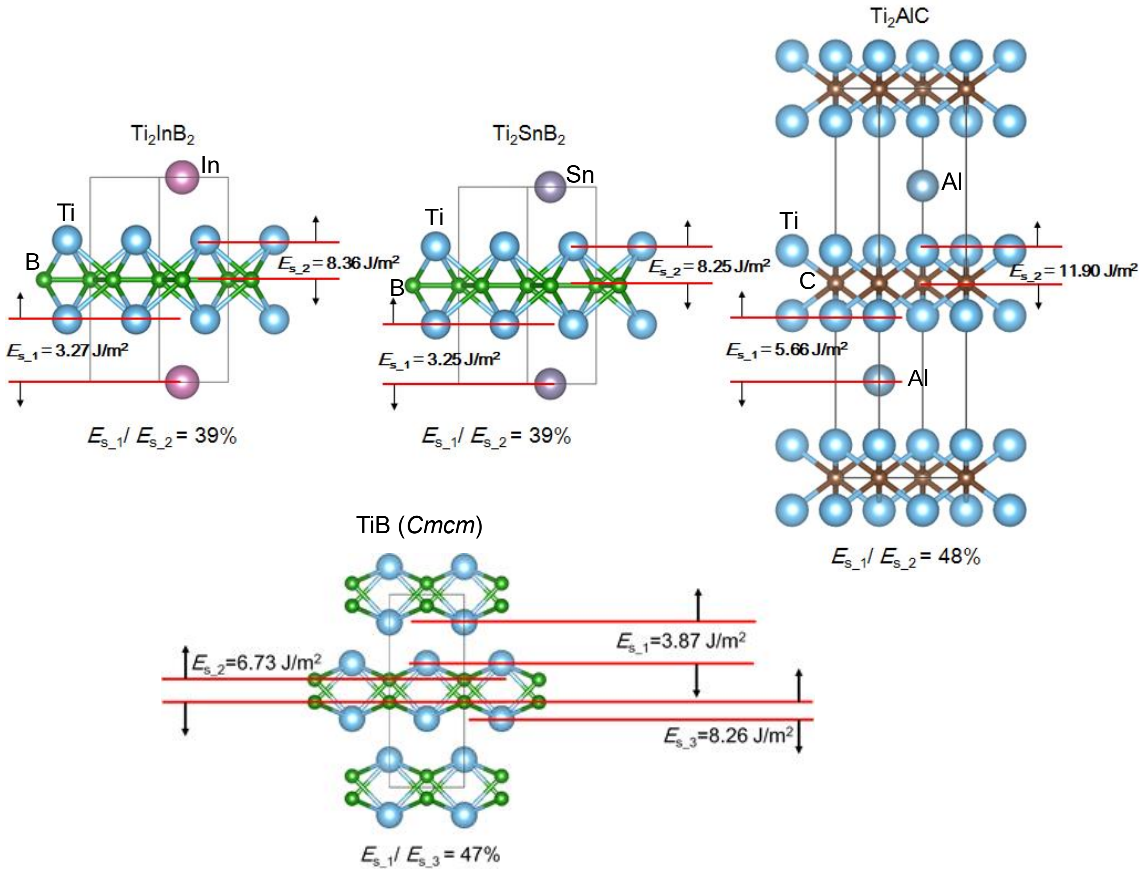

Supplementary Figure 7. Illustration of the separation energies at different interfaces in Ti_2InB_2, Ti_2SnB_2, Ti_2AlC and TiB (*Cmcm*).

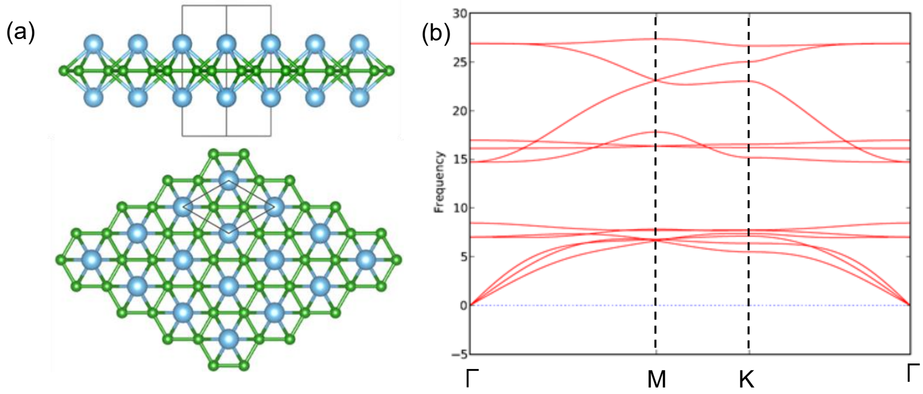

Supplementary Figure 8. Structure (a) and phonon band structure (b) of hexagonal TiB.

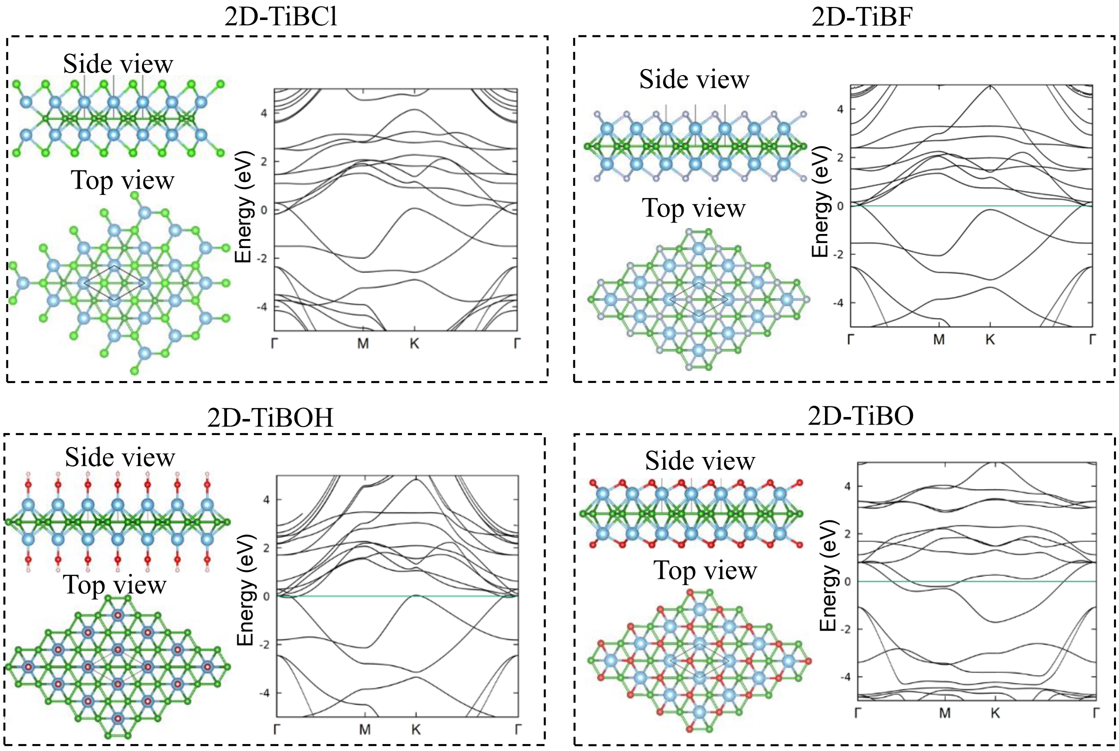

Supplementary Figure 9. Structures and band structures of two dimensional TiB with different functional groups: TiBCl, TiBF, TiBOH and TiBO.

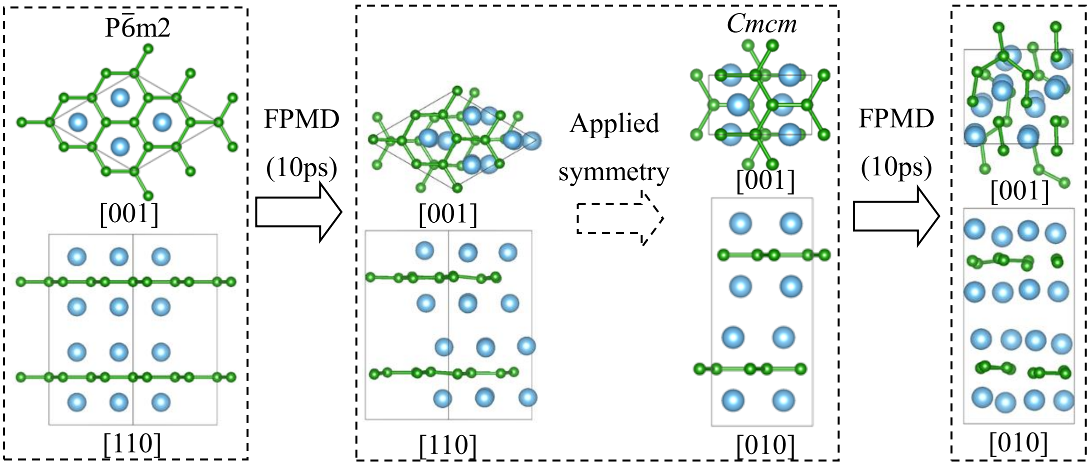

Supplementary Figure 10. First-Principles Molecular Dynamics (FPMD) simulations of layered TiB compounds from hexagonal to orthorhombic structures.

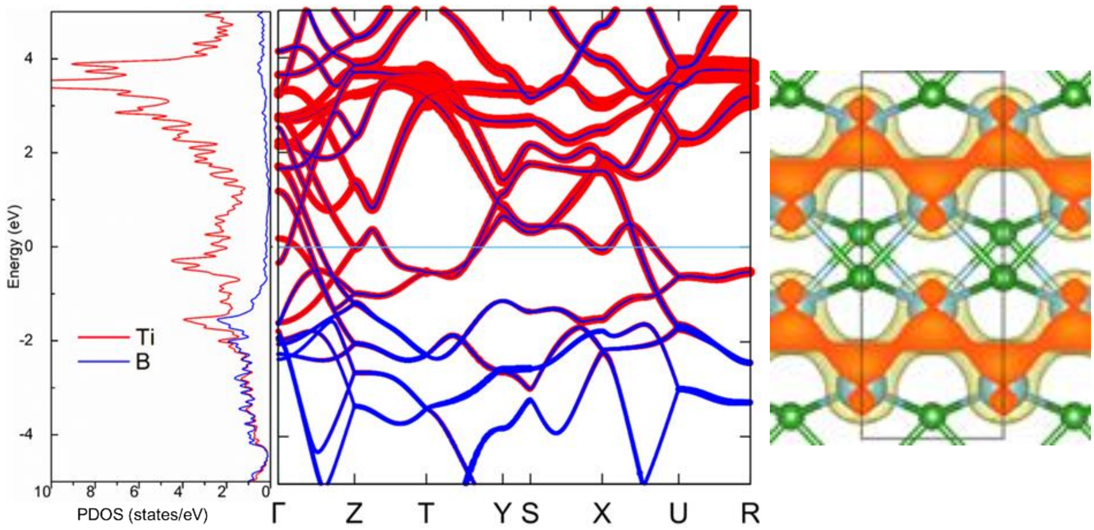

Supplementary Figure 11. The calculated electronic structures of TiB (*Cmcm*). The projected density of states (DOS), projected band structures and electron localization function (ELF) for each structure are shown in the left, central and right regions.

As with the parent Ti_2InB_2 compound, in TiB (*Cmcm*), the hybridization between the *d* orbitals of Ti atoms and *p* orbitals of B atoms is also observed that the bonding (antibonding) states locate below (above) Fermi level and nonbonding Ti states, which locates around Fermi level, form 2-D electron gas at the interfaces between Ti layers (right panel of Fig. S11).

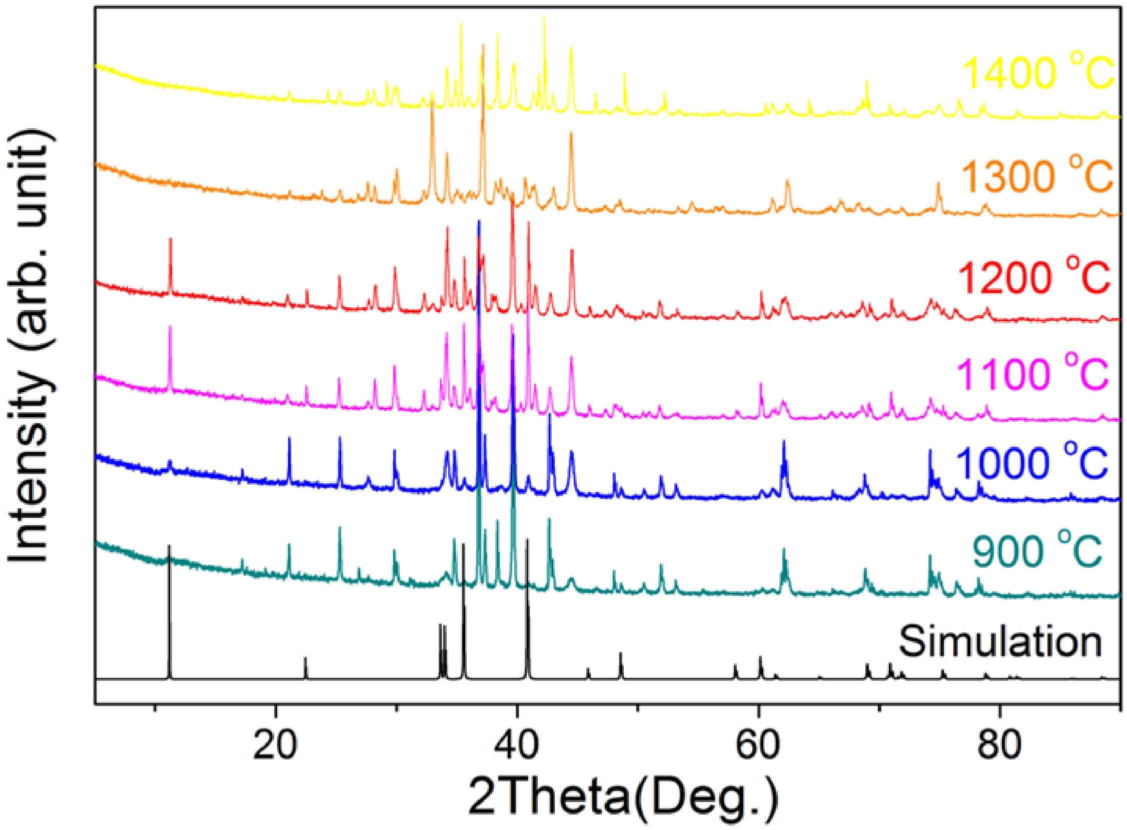

Supplementary Figure 12. Powder XRD patterns showing temperature effect on the synthesis of Ti_2InB_2. The solid state reactions were performed in a tube furnace with Ar gas flow for 10 hours. The starting composition ratio of the elements is stoichiometric.

The huge difference of the boiling point between In (2072 °C) and Ti (3287 ºC)/B (3927 ºC) is an important clue that the compound cannot be grown from the melt. Therefore any trial of high temperature synthesis of pure Ti_2InB_2, including that using arc melting, was failed. Finally a relatively low-temperature method of solid- state reaction route was employed for the synthesis.

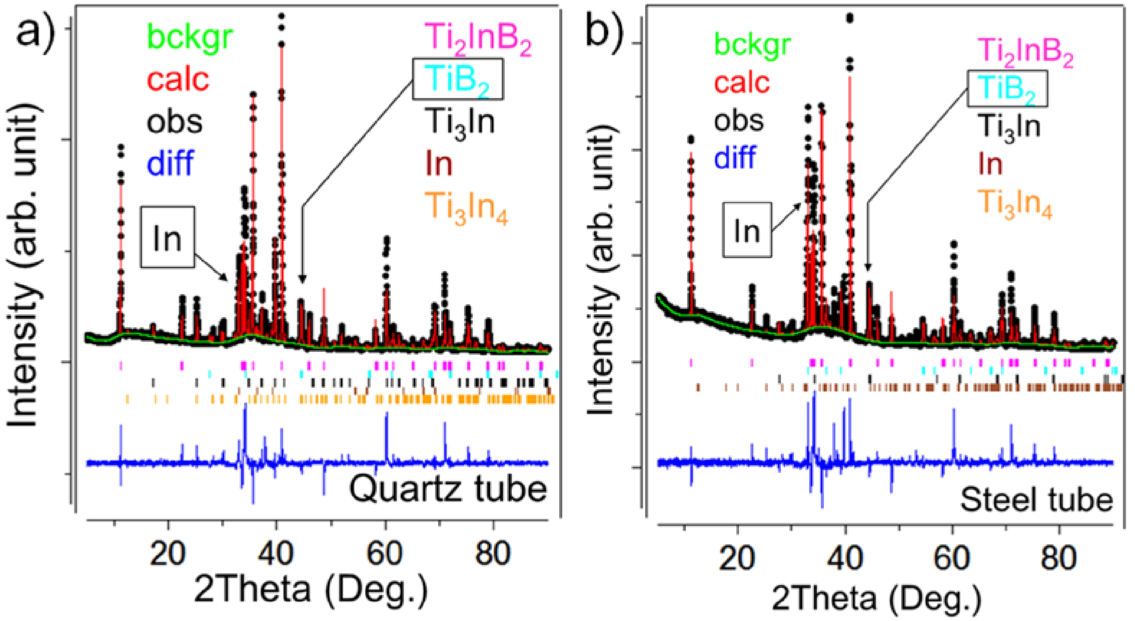

Supplementary Figure 13. Rietveld analyses on the powder XRD patterns of Ti-In-B phases grown by using different crucibles (quartz tube with molybdenum foil as the protector and steel tube respectively). Quartz tube with Mo foil performs better for this reaction as indicated by the higher yield of Ti_2InB_2 and lower yield of In and TiB_2. The composition ratios of the obtained Ti-In-B phases are summarized in Table S2. The reactions were performed on stoichiometric mixtures of Ti, In and B powder at 1100 ^oC for 36 hours. The tubes were sealed with Ar gas inside at 1atm for both cases.

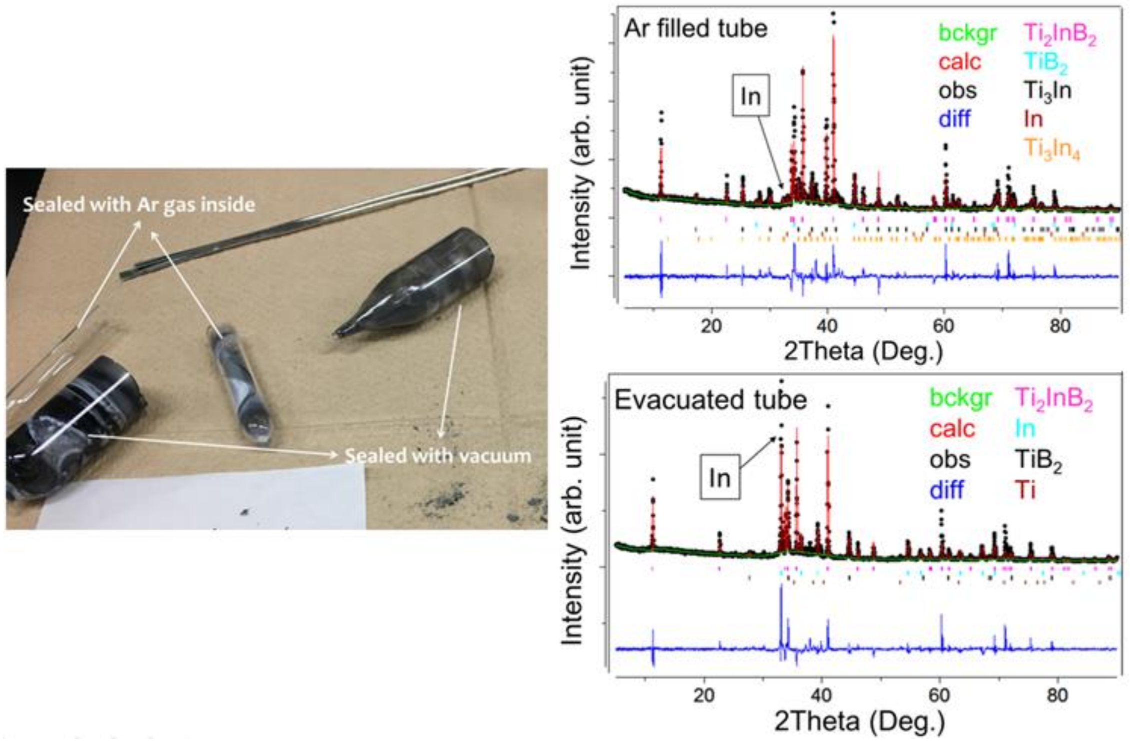

Supplementary Figure 14. The influence of the grown atmosphere (sealed with Ar/vacuum inside) on the synthesis of Ti_2InB_2. Since the triple point of indium is 157 °C and 1kPa, indium easily evaporates under vacuum at elevated temperatures and coats on the inner side of the quartz tube, while the evaporation of indium could be suppressed when Ar gas is filled inside the tube. Rietveld analyses show consistent results. The composition ratios of the obtained Ti-In-B phases are summarized in Table S2. The reactions were performed on stoichiometric mixtures of Ti, In and B powder at 1100 °C for 36 hours. The samples were covered by Mo foil for protection during the reaction.

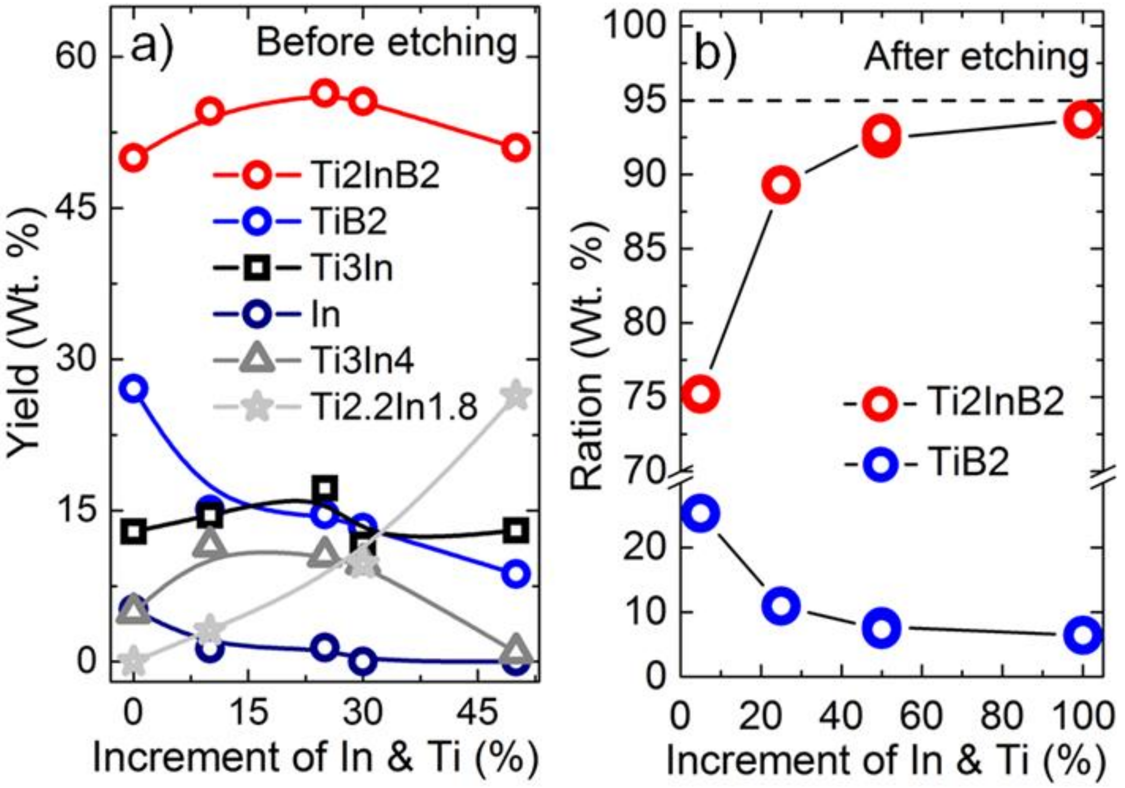

Supplementary Figure 15. The influence of the starting composition ratio of elements. a) Before HCl etching and b) after HCl etching. In order to suppress the formation of TiB_2, which cannot be removed by chemical etching, the amount of Ti and In is increased up to 100%, respectively. The solid state reaction was performed at 1100 °C for 36h under Ar atmosphere, using a sealed quartz tube with Mo foil as the protector. The mass ratio of different phases was obtained by Rietveld analyses of the powder XRD patterns. HCl etching treatment was performed by using 2 mol/L HCl for 10 hours at room temperature.

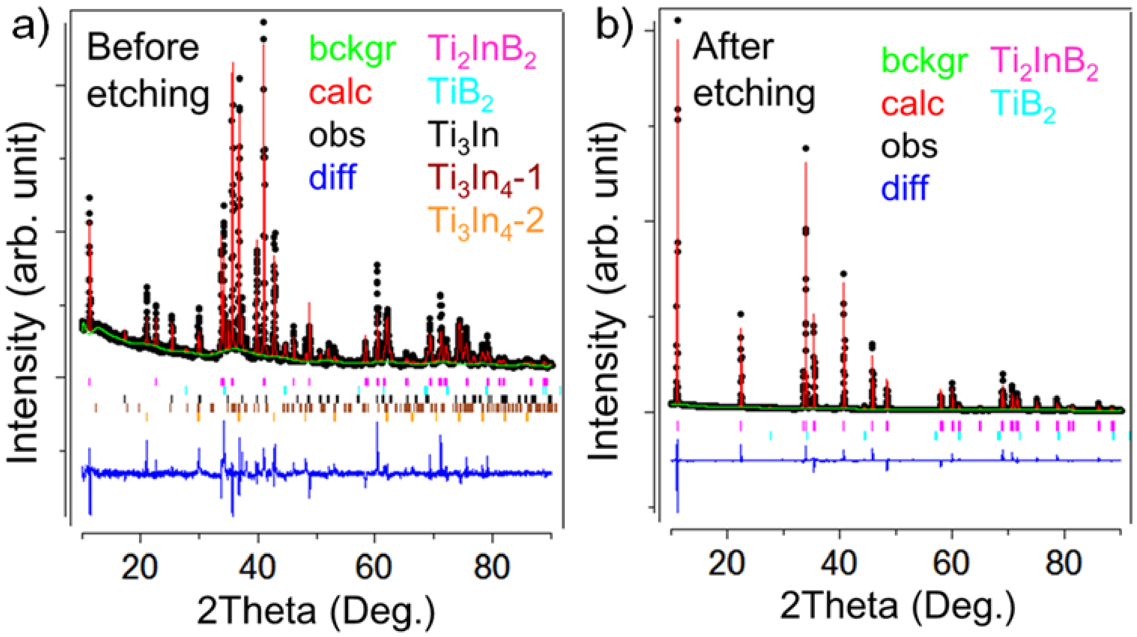

Supplementary Figure 16. Rietveld analyses on the powder XRD patterns of Ti-In-B phases before (a) and after HCl etching (b). After etching all the impurity Ti-In phases are removed, while TiB_2 cannot be removed. The composition ratios of the obtained Ti-In-B phases are summarized in Table S2. The solid state reaction was performed at 1100 °C for 36h under Ar atmosphere, using a sealed quartz tube with Mo foil as the protector. The starting composition ratio of the elements is, Ti:In:B = 3:1.5:2, where the amount of Ti and In were increased by 50% in order to suppress the formation of TiB_2.

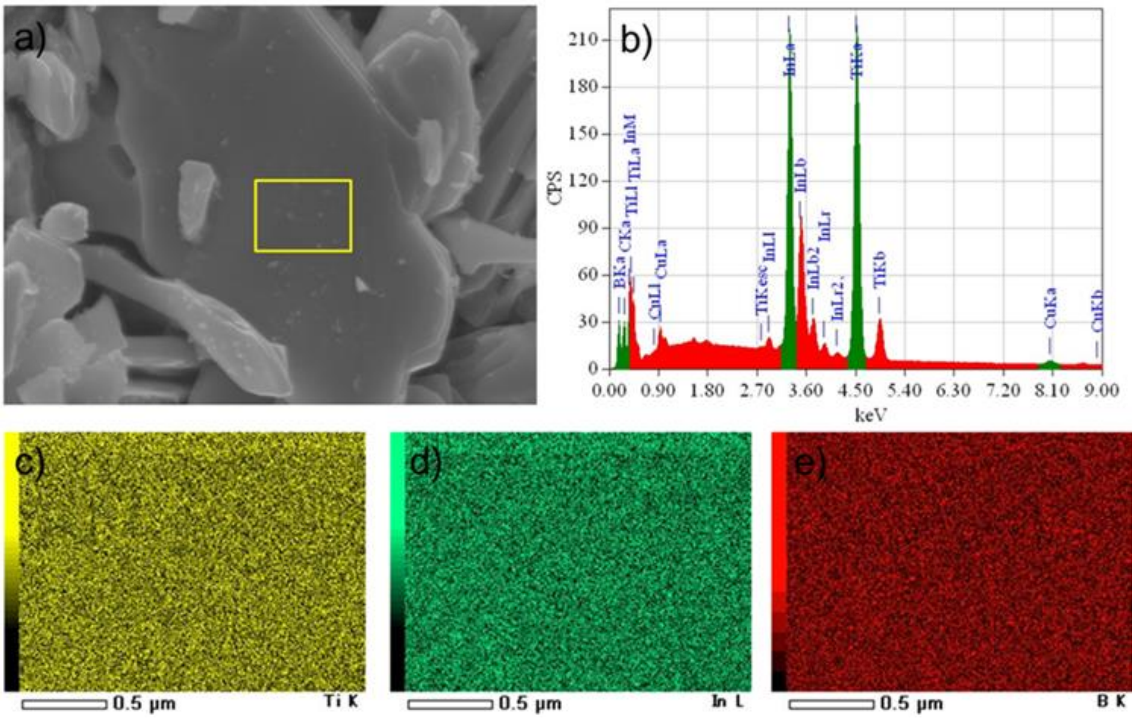

Supplementary Figure 17. SEM image and energy-dispersive X-ray (EDX) spectrum of a grown Ti_2InB_2 sample. a) SEM image of a piece of Ti_2InB_2 particle. The yellow rectangle indicates the area for scanning. b) EDX spectrum for the selected-area scanning. The weak peaks of carbon and copper arise due to the carbon tape and sample stage. The composition ratio was obtained as, Ti:In:B = 1.95:1:2.08, by fixing In as 1. c-e) Element mapping for Ti, In and B, respectively, indicating a homogenous distribution of the component elements.

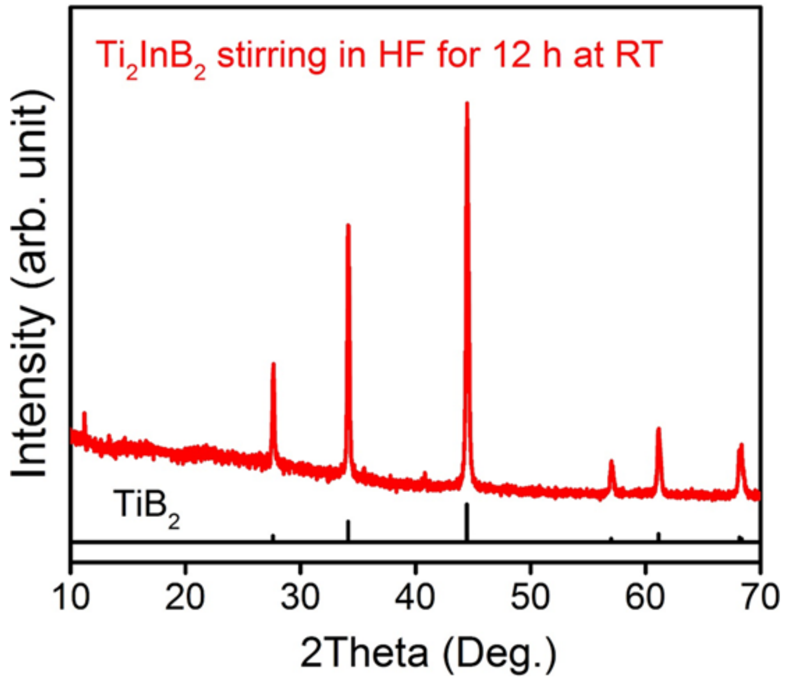

Supplementary Figure 18. XRD pattern of Ti_2InB_2 powders after 50%HF treatment for 12 h at room temperature. After etching in 50%HF, Ti_2InB_2 phases was dissolved first. The residual TiB_2 can be dissolved in excess time. During the total etching process, no new phase was generated, indicating TiB quasi-2D structure cannot be obtained directly by HF etching approach.

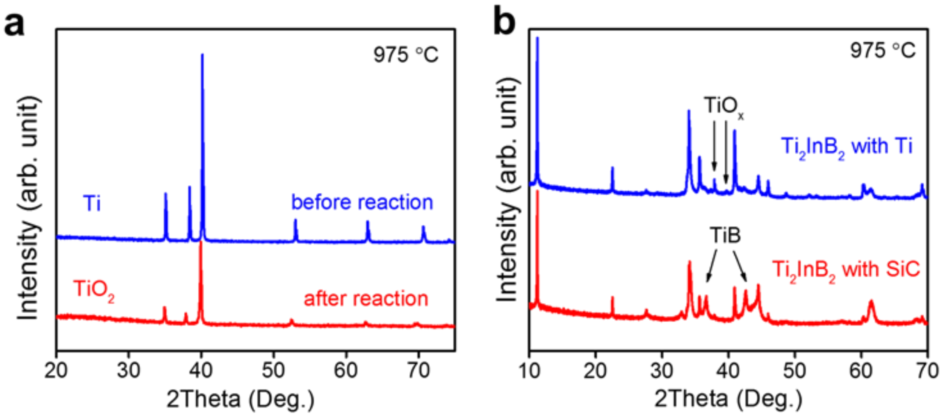

Supplementary Figure 19. Characterization of the oxygen scavenger and products after the dealloying reaction for 6 days at 975 °C under vacuum (~10^{-4} Pa). a, XRD patterns of the oxygen scavenger Ti powders before and after the reaction. b, XRD patterns of Ti_2InB_2 powders after dealloy treatment. The peaks denoted by the black arrows represent new generated phases by using different oxygen scavenger Ti and SiC respectively. In order to prevent the formation of oxides of TiB layers after the removal of In, Ti powders were firstly tried to eliminate the oxygen traces in the reaction

quartz tube. Unfortunately, Ti powders were totally oxidized to TiO_2 during the reaction. Meanwhile, some TiO_x impurities also generated in Ti_2InB_2 sample. When we switch to SiC as the oxygen scavenger, the products shows quite good purity with only TiB phase generate.

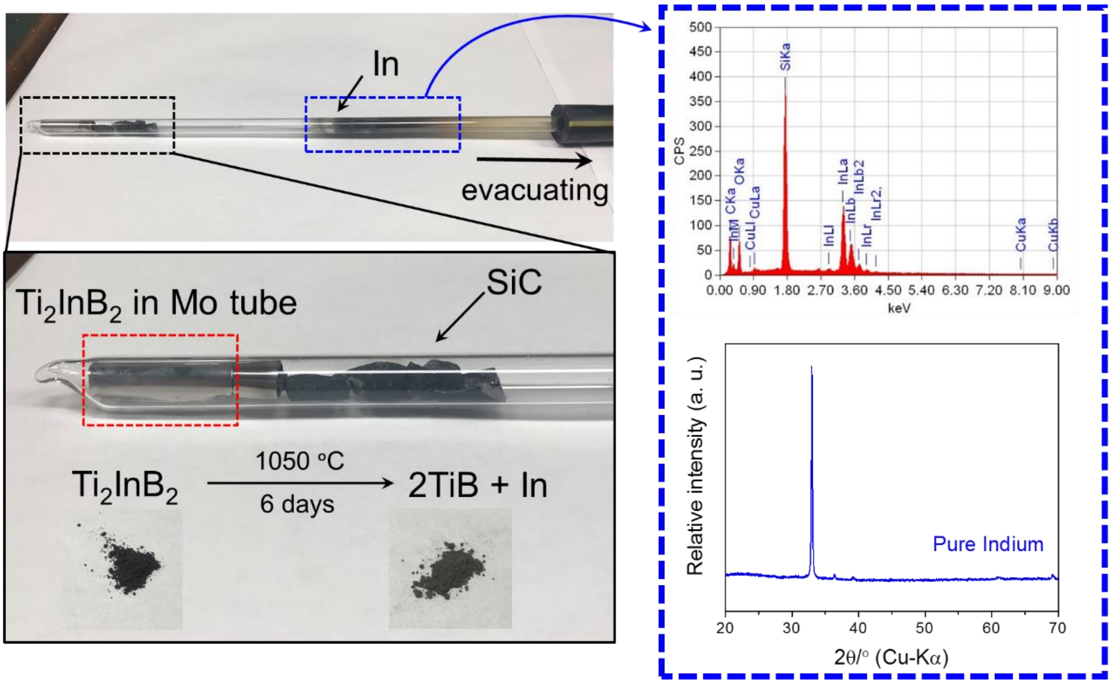

Supplementary Figure 20. Schematic of TiB preparation. First, the as-prepared Ti_2InB_2 powders were filled in a Mo tube. The Mo tube was put into the end of the quartz tube. Second, several SiC ingots were placed in the front of Mo tube as oxygen scavenger. Finally, both of the Mo tube and SiC ingots were heated at 1050 °C (5 °C min^{-1}) for 6 days in the dynamically evacuated quartz tube (~10^{-4} Pa). The reaction process can be described as the equation as below:

$$Ti_2InB_2 \rightarrow 2TiB + In\,(vap.) \quad (1)$$

The obtained product TiB was puce color, different from dark grey of starting materials Ti_2InB_2.

EDS (upper) and XRD (lower) spectra in blue rectangle indicates that the formed metal species in the inner wall of the quartz tube during the evacuation process was In metal.

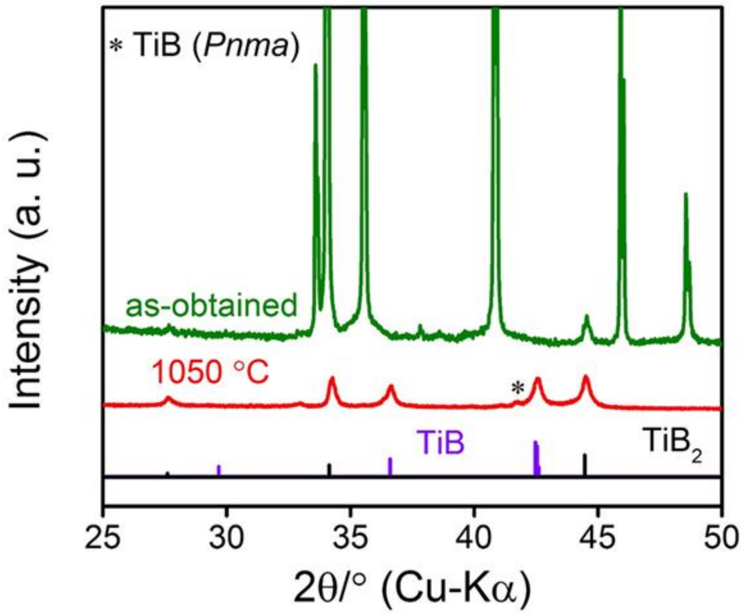

Supplementary Figure 21. Enlarged XRD patterns of the Ti_2InB_2 sample before and after dealloying treatment.

FPMD simulations.

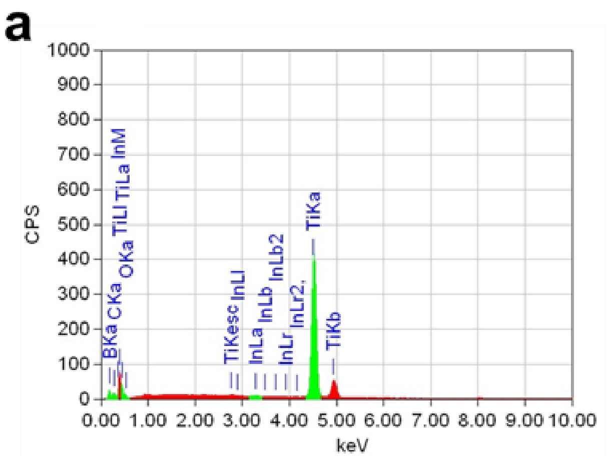

Elements (KeV)	Atom%
B 0.183	39.18
C 0.277	9.65
O 0.525	12.85
Ti 4.508	38.22
In 3.285	0.10
Total	100

Supplementary Figure 22. EDS characterization of TiB. a, The EDS spectrum of product after the dealloying reaction for 6 days at 1050 °C under vacuum (~10^{-4} Pa), which contains B, C, O, Ti and negligible In species. b, The atomic ratios determined by EDS spectrum. Since almost no In peaks were observed in the EDS pattern, it is reasonable to conclude that all of the In was extracted during the dealloying process. The carbon signals in EDS results mainly originated from the carbon tape that we used to attach the powder sample onto the sample holder (copper stage). And the oxygen species

should be the adsorbed oxygen molecule from the air because the TiB sample was exposed and stored in the air.

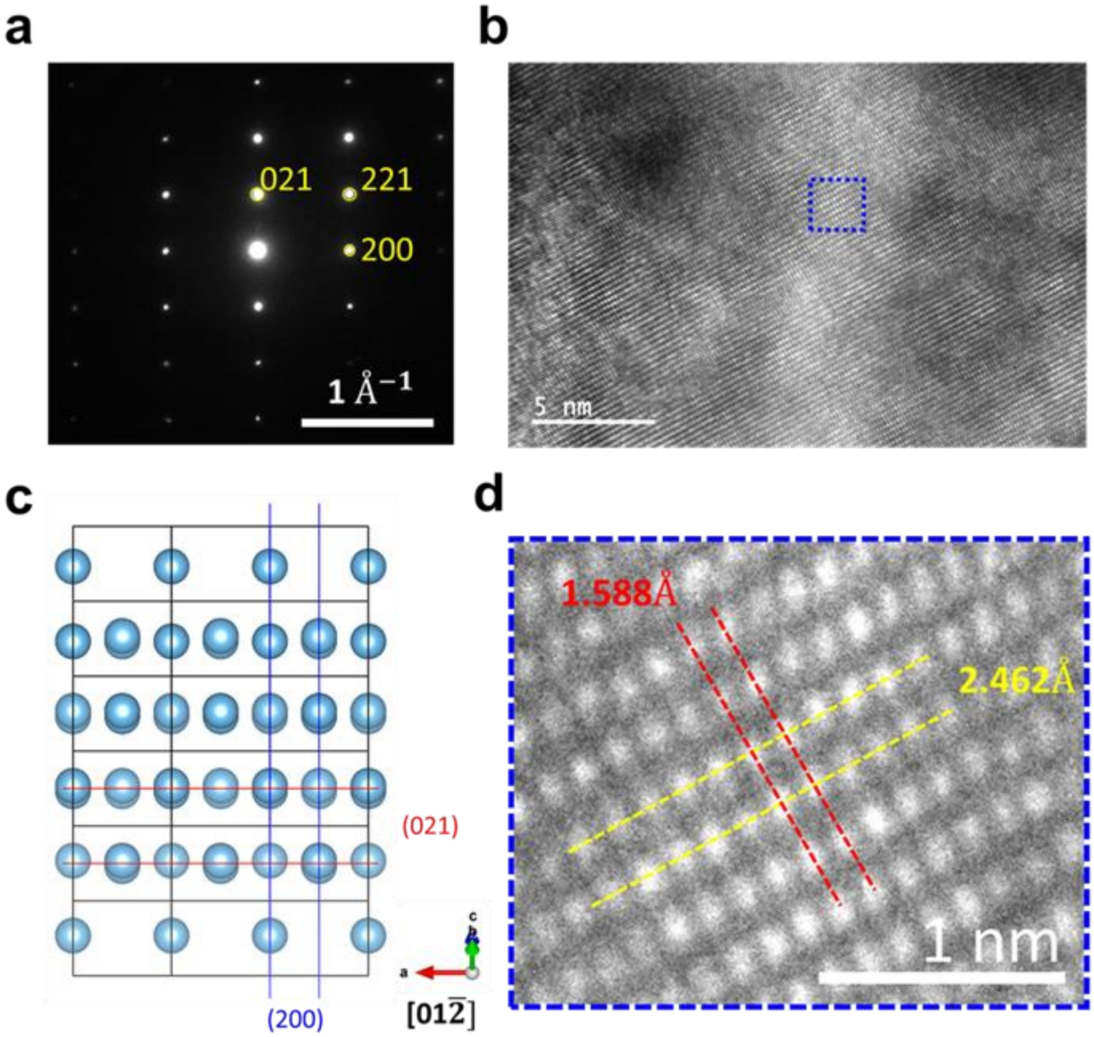

Supplementary Figure 23. Structurel characterization of TiB. a, Overall selected area electron diffraction pattern of the TiB flake along [01$\bar{2}$] direction. b, Corresponding atom resolved HRTEM image of TiB phase along [01$\bar{2}$] direction. c, Simulated crystal structure of TiB phase with orthorhombic group (*Cmcm*) along [01$\bar{2}$] direction, the blue balls represent Ti atoms. Here, the B atoms were hidden to facilitate the comparison with the HRTEM image, which is due to the low scattering intensity of B elements. d, Enlarged HRTEM image from b and the affixed interlayer space of (021) and (200) plane. The interplanar spacing of (021) and (200) were also obtained to be 2.462 Å and 1.588 Å, respectively, in agreement with simulation results.

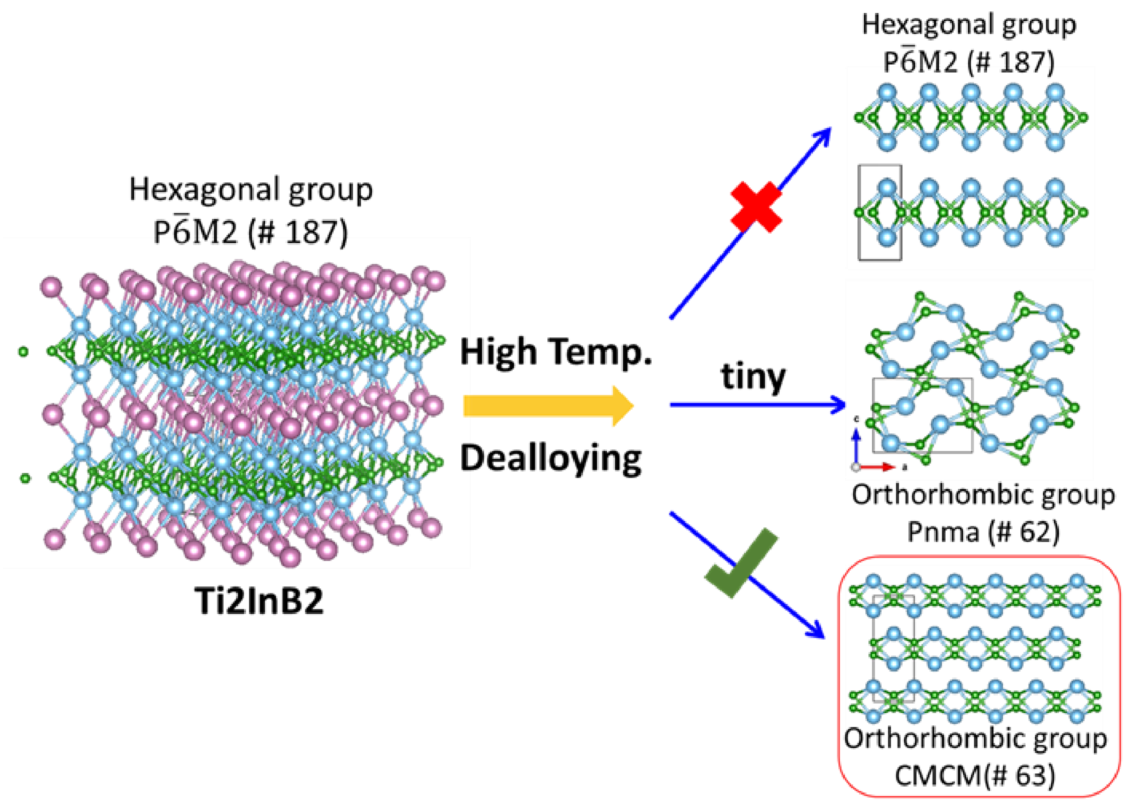

Supplementary Figure 24. Proposed the generated crystal structures from the parent Ti_2InB_2 MAX phase. From the XRD pattern of the dealloyed product, the TiB phase with hexagonal group *(P$\bar{6}$m2*) can not be obtained due to the harsh reaction condition. Instead, another stable layered TiB phase with orthorhombic group (*Cmcm*) was generated. At the same time, tiny TiB phase with another orthorhombic group (*Pnma*) was also generated (Fig. S25).

Our calculations (Table S1) show that the stability of layered TiB phase with orthorhombic symmetry (*Cmcm*) and another orthorhombic TiB structure (*Pnma*) are comparable and is slightly more stable than the structure with hexagonal group (*P$\bar{6}$m2*). It was intuitive to expect that the removal of In atoms from Ti_2InB_2 could directly produce hexagonal TiB (Fig. S8). However, local displacements of Ti and B atoms motivated by the high temperature led to a generation of orthorhombic (*Cmcm*) phase with layered structure as shown in Fig. S10. Therefore, we consider the obtained orthorhombic phase was originated from a phase change of hexagonal (*P$\bar{6}$m2*) with the similar layered structure. The layered structure of TiB is kept after the hexagonal-to-orthorhombic phase change. Moreover, the difference of the calculated energies of the two TiB structures with orthorhombic symmetry *Cmcm* and *Pnma* are quite small (Fig. S25 and Table S1). This means that these two orthorhombic structures possess similar thermodynamic stabilities. However, a significant energy barrier can be expected for the phase change from layered structures (P$\bar{6}$m2 and *Cmcm*) to 3D *Pnma* structure. Therefore, the formation of TiB with *Pnma* symmetry is not dynamically favored. Consequently, the enlarged XRD pattern (Figure S21) of

1050 °C obtained sample can only give a tiny TiB with *Pnma* space group.

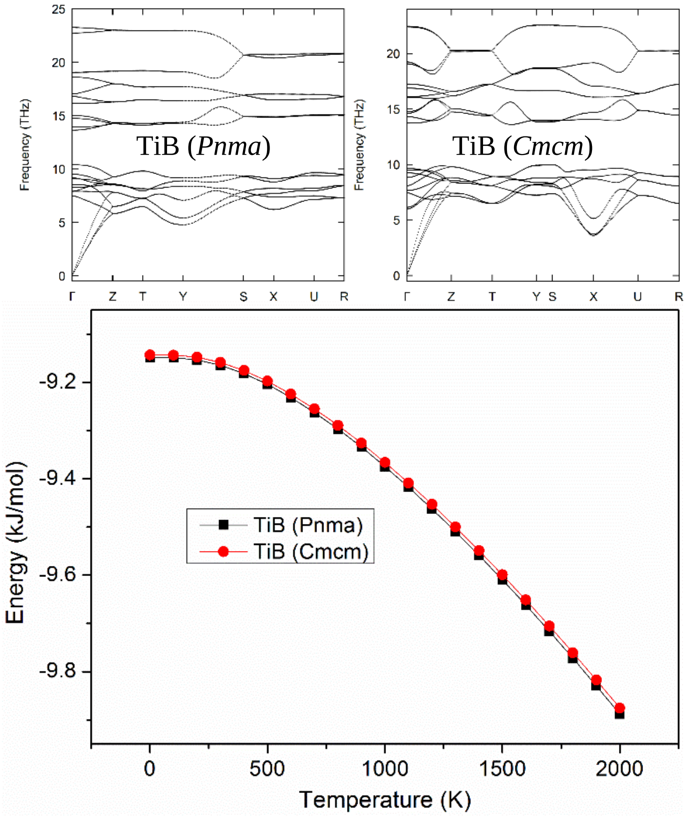

Supplementary Figure 25. The computed phonon band structures and Helmholtz free energies of the two TiB compounds with symmetries of *Pnma* and *Cmcm* at ambient pressure.

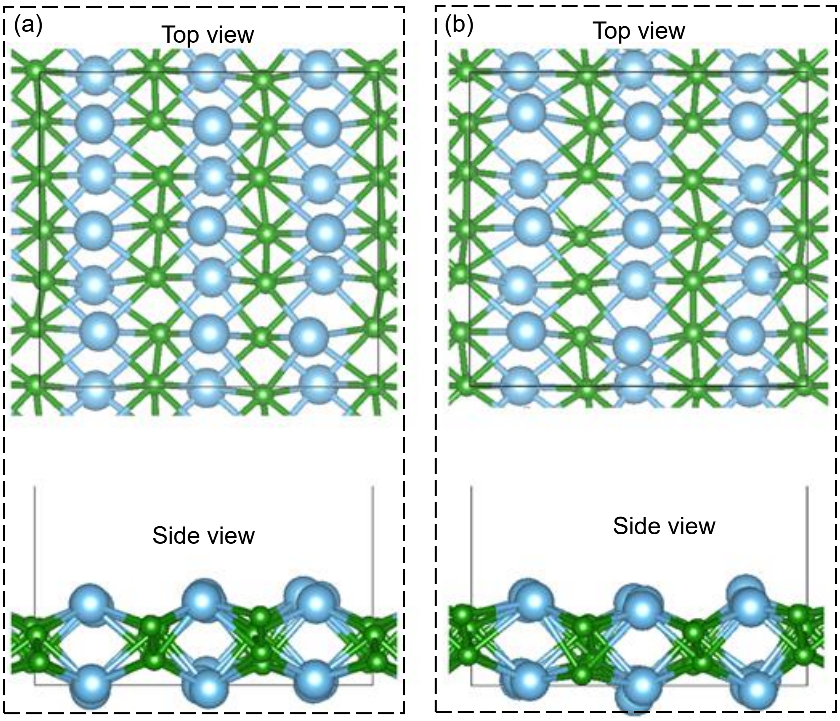

Supplementary Figure 26. The equilibrium structures of 2D-TiB (*Cmcm*) at 1273 K (a) and 1773 K (b) after 10 ps.

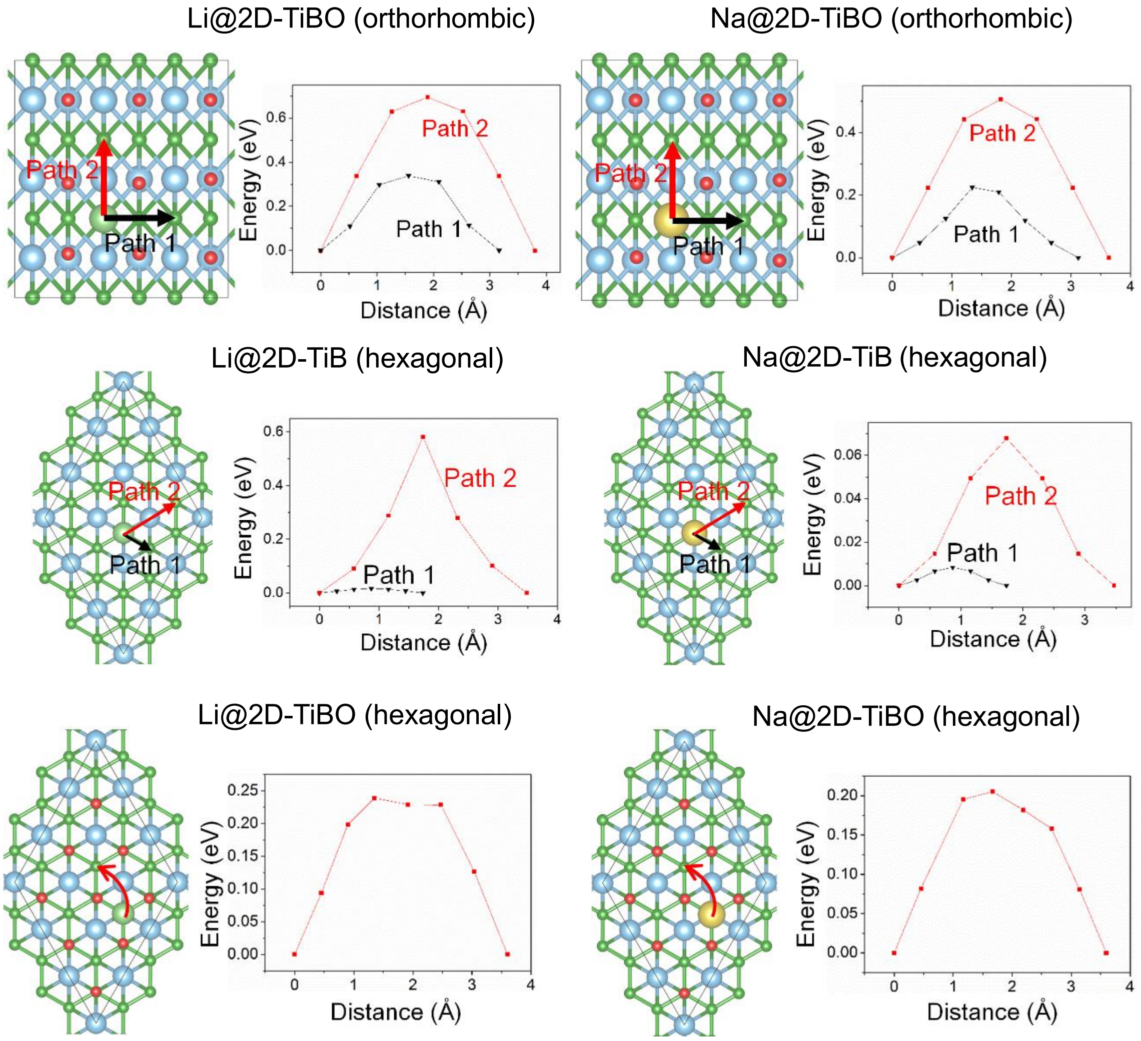

Supplementary Figure 27. Diffusion behavior of Li and Na on 2D- TiBO (orthorhombic and hexagonal) and TiB (hexagonal).

Supplementary Table 1. Space group, atomic position, calculated energy and lattice parameters of TiB, Ti_2InB_2 and Ti_2SnB_2.

Compound	Space group	Atomic position	Energy (eV/atom)	Lattice parameter (Å)		
				a	b	c
TiB	*Cmcm*	Ti (0.5000, 0.6431, 0.7500)<br>B (0.0000, 0.5607, 0.2500)	-9.2177	3.2199	8.3627	3.0253
	Pnma	Ti (0.6776, 0.2500, 0.7628)<br>B (0.0297, 0.2500, 0.5992)	-9.2231	6.1188	3.0543	4.5659
	$P\bar{6}m2$	Ti (0.3333, 0.6667, 0.3783)<br>B (0.0000, 0.0000, 0.5000)<br>B (0. 6667, 0. 3333, 0.5000)	-9.0778	3.0011	3.0011	5.8937

Ti_2InB_2	$P\bar{6}m2$	Ti (0.3333, 0.6667, 0.6935)<br>In (0.6667, 0.3333, 0.0000)<br>B (0.0000, 0.0000, 0.5000)<br>B (0. 6667, 0. 3333, 0.5000)	-7.9620	3.0771	3.0771	7.9172
Ti_2SnB_2<br>(10GPa)	$P\bar{6}m2$	Ti (0.3333, 0.6667, 0.6982)<br>Sn (0.6667, 0.3333, 0.0000)<br>B (0.0000, 0.0000, 0.5000)<br>B (0. 6667, 0. 3333, 0.5000)	-8.2653	3.1020	3.1020	7.7114

Supplementary Table 2. Calculated values of bulk modulus B (in GPa), shear modulus G (in GPa), anisotropic Young's moduli *E* (in GPa) and Poisson's ratio *v* of Ti_2InB_2, Ti_2AlC and Ti_3AlC_2 in comparison with the experimental data of Ti_3AlC_2.

Compound	*B*	*G*	*E*	*v*
Ti_2InB_2	147	122	E_x=E_y=337<br>E_z=256	v_{xy}=v_{yx}=0.12<br>v_{xz}=v_{yz}=0.19<br>v_{zx}=v_{zy}=0.15
Ti_2AlC	130	115	E_x=E_y=282<br>E_z=252	v_{xy}=v_{yx}=0.14<br>v_{xz}=v_{yz}=0.17<br>v_{zx}=v_{zy}=0.15
Ti_3AlC_2	155	132	E_x=E_y=326<br>E_z=276	v_{xy}=v_{yx}=0.16<br>v_{xz}=v_{yz}=0.19<br>v_{zx}=v_{zy}=0.16
Ti_3AlC_2<br>(experiment*)	165	124	298	0.20

*M. W. Barsoum, Prog. Solid State Chem. 28, 201 (2000).

Supplementary Table 3. Solid-state reaction route to increase the yield of Ti_2InB_2 while decrease the yield of TiB_2.

No.	Synthesis condition[a]	Weight ratio (wt. %) obtained by Rietveld analysis						Remarks
		Ti_2InB_2	TiB_2	Ti_3In	In	Ti_3In_4	Other	
1	SUS[b], 1100 °C, 36h, Ar	48.2	33.0	5.4	13.3	1.9	U[d]	**Crucible Effect**
2	QM[c], 1100 °C, 36h, Ar	50.0	27.1	12.9	5.2	4.9	U	
3	QM, 1100 °C, 60h, Vac.	43.0	37.6	0	19.4	0	U	**Atmosphere effect**
4	QM, 1100 °C, 60h, Ar	46.1	26.4	19.6	0.8	7.2	U	
2	QM, 1100 °C, 36h, Ar	50.0	27.1	12.9	5.2	4.9	U	**Annealing effect 1: at 1100 °C**
2.1	QM, 1100 °C, 100h, Ar	50.0	34.8	0	10.3	4.2	U	
2.2[e]	QM, 1100 °C, 36h, Ar	43.9	37.6	0	13.9	4.6	U	
1	SUS, 1100 °C, 36h, Ar	48.2	33.0	5.4	13.3	1.9	U	**Annealing effect 2: at different temperatures**
1.1	SUS, 1100 °C, 100h, Ar	36.2	44.1	0	16.2	3.4	U	
1.2[f]	QM, 1200 °C, 12h, Ar	0	√	-	√	-	Ti_2In_5	
1.3[f]	QM, 1100 °C, 36h, Ar	14.4	56.8	0	28.8	0	U	
1.4[f]	QM, 1050 °C, 36h, Ar	30.9	44.8	0	24.3	0	U	
1.5[f]	QM, 1000 °C, 168h, Ar	31.1	45.4	0	23.4	0	U	
5	QM, 1100 °C, 5h, Ar	37.8	26.6	20.7	1.6	13.3	U	**Annealing Effect 3: short time**
5.1[g]	QM, 1100 °C, 5h, Ar	37.6	33.8	11.2	10.4	7.1	U	
5.2[h]	QM, 1100 °C, 5h, Ar	39.7	37.2	1.0	15.1	7.1	U	
5.3[i]	QM, 1100 °C, 5h, Ar	31.9	39.4	0.8	20.8	7.1	U	
6	Specified[j], Ti (+15%)	53.0	16.1	12.9	0	3.1	$Ti_{2.2}In_{1.8}$	**The influence of initial composition ratio**
7	Specified, In (+5%)	50.2	22.6	13.8	2.8	10.6	U	
8	Specified, In (-5%)	47.7	24.2	15.5	1.3	11.2	U	
9	Specified, B (+10%)	41.2	30.9	13.5	5.0	9.3	U	
10	Specified, Ti (+30%)	27.1	17.9	33.2	0	1.0	$Ti_{2.2}In_{1.8}$	
11	Specified, In (+50%)	38.0	26.5	10.4	23.0	2.0	U	
12	Specified, Ti-In (+10%)	**54.6**	**15.1**	14.5	1.3	11.5	$Ti_{2.2}In_{1.8}$	
13	Specified, Ti-In (+25%)	**56.4**	**14.6**	17.2	1.4	10.5	$Ti_{2.2}In_{1.8}$	
14	Specified, Ti-In (+30%)	**55.6**	**13.3**	11.6	0	9.8	$Ti_{2.2}In_{1.8}$	
15	Specified, Ti-In (+50%)	**51.0**	**8.7**	13.0	0	0.93	$Ti_{2.2}In_{1.8}$	

a. If not specified, the starting composition ratio of Ti, In and B is stoichiometric;

b. SUS: stainless steel tube; c. QM: quartz tube with Mo foil as the protector; d. U: unknown phases; Annealing effect, e: the annealing was made based on 2.1; f: the annealing was made based on 1.1; g,h,i: the annealing was made based on 5, 5.1, and 5.2, respectively. Before annealing, the products

were crushed and mixed again for homogeneity; j: The specified condition is, “QM, 1100 ^{o}C, 36h, Ar”.
